# Incorporation of Phosphatase Inhibitor in Culture Prompts Growth Initiation of Isolated Non-Growing Oocytes

**DOI:** 10.1371/journal.pone.0077533

**Published:** 2013-11-04

**Authors:** Kanako Morohaku, Yumi Hoshino, Hiroshi Sasada, Eimei Sato

**Affiliations:** 1 Laboratory of Animal Reproduction, Graduate School of Agriculture Science, Tohoku University, Sendai, Japan; 2 Laboratory of Animal Reproduction, School of Veterinary Medicine, Kitasato University, Towada, Japan; Baylor College of Medicine, United States of America

## Abstract

*In vitro* folliculogenesis of primordial and early preantral follicles is necessary for increment of reproductive efficiency in domestic animals, humans and endangered species. Recent study in phosphatase and tensin homolog (*Pten*) -knockout mice has revealed that this phosphatase acts as an inhibitory factor in follicle activation of primordial pool with the resultant inhibition of oocyte growth. To test *in vitro* effect of a phosphatase inhibitor on growth initiation of isolated non-growing oocytes in neonatal ovaries, we applied a specific inhibitor (bpV (HOpic)) for PTEN in culturing system. Non-growing oocytes isolated from the ovaries of newborn BDF1 (C57BL/6 × DBA/2) pups were divided to four culture groups. Five days after culture, the oocytes in 14 μmol/l bpV only, 14 μmol/l bpV plus 100 ng/ml Kit Ligand (KL), and 100 ng/ml KL groups showed significantly (*P*<0.05) growth (19.3±0.55, 25.8±0.53 and 21.6±0.29 μm, respectively) compared with that of the control (no additive) (16.9±0.53 μm). In addition, western blotting in those groups showed enhanced expression of phosphorylated Akt. In conclusion, we clearly demonstrate that isolated non-growing oocytes develop in phosphatase inhibitor, especially to PTEN, incorporated culturing system, and show first as we know that oocytes with zona Pellucidae can be obtained *in vitro* from isolated non-growing oocytes.

## Introduction

In the mammalian ovaries, several to ten thousands of primordial follicle’s oocytes (non-growing primary oocytes) are contained at birth, and these oocytes situate in the ovarian cortex in long duration of the fertile period [1–3]. Up to now, for increment of reproductive efficiency in domestic animals, humans and endangered species, many studies have been attempted to develop techniques to use the ovarian oocytes. Of these, some techniques such as superovulation with hormone treatment or *in vitro* culture can allow us to use more oocytes from late preantral and antral follicles [4–7], but with a limited. On the other hands, for purpose of utilization of the whole ovarian follicles, *in vitro* organ culture of neonatal ovaries in mice has succeeded in production of pups by the following *in vitro* fertilization and embryo transfer of matured oocytes derived from primordial follicles, but with only a few reports [8,9]. Thus, the ensure method for use of ovarian oocytes, especially in primordial follicles that are present as a stock of primary oocytes in the ovary remains to be developed.

Primordial follicles start growing with “follicle growth initiation or follicle activation” from primordial follicle pool, substantially evaluated by growth initiation of primary oocytes included, which is termed as the entry of primordial follicles into primary follicle stage [1,3]. This activation is indispensable for oocyte growth. Recent study in *Pten* (phosphatase and tensin homolog) -knockout mice [10] has revealed that this phosphatase acts as an inhibitory factor in follicle activation of primordial follicle pool. A lipid phosphatase PTEN dephosphorylates the 3 position of the inositol ring of inositol phospholipids as a major negative regulator of PI3K that is a fundamental signaling for the regulation of cell proliferation, survival, migration, and metabolism through the Akt pathway [11–13]. Considered together that the study of PTEN knockout mouse showed precocious follicle development of all primordial follicles, it positively motivated us to develop a method feasible for more efficient growth initiation of primary oocytes by incorporation of PTEN inhibitor in culture media. 

To demonstrate *in vitro* growth of primary oocytes, this study was aimed to examine *in vitro* growth of isolated non-growing oocytes in culture media with a specific inhibitor bpV (HOpic) for PTEN [14]. Although *in vitro* organ or slice culture of mouse ovaries succeeded in growth of primordial follicles, these methods look like unfeasible for large ovaries [1]. On the other hand, a method for culture of isolated non-growing primary oocytes would be expected to be applied independent of size of the ovary. In this study, we show first as we know that phosphatase inhibitor, especially to PTEN can stimulate *in vitro* growth activation of isolated non-growing primary oocytes and that more effectively concomitant with KL the oocytes can be grown with formation of their surrounding zona pellucidae. On this point, since non-growing oocytes are stored in vivo as individual follicle and grown accompanied by follicle development, it is known that oocyte growth is sustained by its surrounding cells (granulosa cells), and also that a relationship between an oocyte and its accompanied cells differs according to follicle developmental stage, in which initial growth of non-growing oocytes is sustained by humoral factors rather than granulosa cells. Consequently, we believe that our study shows possibility and significant step of in vitro cell-free culture for non-growing primary oocytes. 

## Materials and Methods

### Animal care and use

BDF1 (C57BL6×DBA) female mice were used throughout the experiments and were maintained under the specific pathogen -free in 14 hours light -10 hours dark controlled light condition. The present study was approved by Ethical Committee of Tohoku University. 

### Isolation of non-growing oocytes

The ovaries of newborn pups were collected at day 0 (day of delivery) using fine forceps and removed extra tissues with 26G needles (Terumo, Tokyo, Japan) in pre-warmed Leibovitz’s L-15 medium (GIBCO, Invitrogen, CA, USA) containing 5% (v/v) fetal bovine serum (FBS) (Benchmark, Gemini Bio-Products, CA, USA). After washing ovaries with fresh Leibovitz’s L-15 medium, the ovaries were surgically minced with 32G needles (Misawa Medical Co, Tokyo, Japan) in Minimum Essential Medium Alfa (α-MEM, GIBCO, Invitrogen, CA, USA) containing 5% (v/v) FBS to isolate non-growing oocytes. Then, only the oocytes having even surface were selected and washed three times in α-MEM containing 5% (v/v) FBS with a glass pipette (Sutter Instrument, CA, USA) of 30 μm in diameter. All procedures for isolation to selection of non-growing oocytes were performed under the dissection microscope (Olympus Co, Tokyo, Japan) at 37°C with warm plate (Narishige, Mishima, Japan).

### In Vitro culture of non-growing oocytes

In experiment 1, to examine effect of phosphatase inhibitor on *in vitro* growth, the oocytes prepared were at random divided to two culture groups: no additive (control) and phosphatase inhibitor (PhosSTOP, Roche Diagnostics K.K., Tokyo, Japan) supplemented α-MEM containing 5% (v/v) FBS, 1% (v/v) ITS (Insulin/transferrin/serine, GIBCO, Invitrogen, CA, USA) and 100 mIU/ml FSH (Sigma-Aldrich Japan, Tokyo, Japan). PhosSTOP tablet was dissolved in culture medium according to the instruction of the supplier. In experiment 2, to demonstrate effect of a specific inhibitor of PTEN on *in vitro* growth of non-growing oocytes, they were cultured in α-MEM containing 5% (v/v) FBS, 1% (v/v) ITS and 100 mIU/ml FSH supplemented with either of 0, 0.14, 1.4, 14, or 140 μmol/l bpV (HOpic) (Dipotassium bisperoxo (5-hydroxypyridine-2-carboxyl)-oxovanadate, Alexis Biochemicals, CA, USA) by referring the previous report [10,15]. In experiment 3, to demonstrate synergic effect of a specific inhibitor of PTEN and KL on *in vitro* growth of non-growing oocytes, they were cultured in α-MEM containing 5% (v/v) FBS, 1% (v/v) ITS and 100 mIU/ml FSH supplemented with either of 14 μmol/l bpV only, 14 μmol/l bpV plus 100 ng/ml KL (Sigma-Aldrich Japan, Tokyo, Japan), 100 ng/ml KL only, or no additive. Before this experiment, the preliminary experiment was carried out to determine the concentration of KL effective for *in vitro* growth of the oocytes (data not shown). In each experiment, single oocytes were cultured in 5 μl droplets covered with 5 μl mineral oil (Nakarai tesque Co., Kyoto, Japan) in HLA plate (Greiner bio-one, Tokyo, Japan) in CO_2_ incubator (Hirasawa Co., Tokyo, Japan) under highly humidified atmosphere of 5% CO_2_ in air at 37°C. Before starting culture, the droplets were pre-equilibrated for 3-4 hours under the same condition. The morphology and *in vitro* growth of each oocyte were observed every day and evaluated as normality of its shape and color, and the size by measuring two perpendicular diameters with a microscope (IMT2) (Olympus Co., Tokyo, Japan) attached with an optic micrometer. In the case, where the oocytes showed decrease in their diameter and uneven surface with darkness, they were classified as degenerating ones. The data in each experiment were accumulated from at least 3 times replications.

### Histological analysis

For histological examination basically according to the previous reports [16,17], the samples were pre-embedded in 2% (w/v) agar (Nippon gene, Tokyo, Japan) and fixed with 10% (v/v) formaldehyde (Wako Co., Tokyo, Japan) in phosphate buffered saline (PBS) (Wako Co., Tokyo, Japan) up to 48 hours at room temperature. After dehydration with alcohol, they were embedded in paraffin at 60°C. Paraffinized samples were sectioned at 7 μm by LEICA RM 2065 (Leica Geosystems Co., Tokyo, Japan), and the sections were stained with hematoxylin and eosin. For staining isolated oocytes, they were stained with Hoechst 33342 (Sigma-Aldrich Japan, Tokyo, Japan) for 3 minutes under warm condition at 37°C and subjected to fluorescent observation under microscope.

For immunostaining of mouse vasa homologue (MVH), the deparaffinized sections were at first treated for 20 minutes with 3% (v/v) hydrogen peroxide dissolved in pure methanol to block endogenous peroxidase activity and then incubated for 20 minutes with 10% (v/v) normal goat serum to saturate nonspecific binding sites for immunoglobulin G (IgG). The sections were next incubated in anti-MVH serum (Abcam Inc., MA, USA) diluted at 1:200 for overnight at 4°C. After each step, the sections were washed two times for 5 minutes in PBS. Visualization of peroxidase activity was performed using DAB kit (Vector Laboratories Inc., CA, USA).

### Western blotting

For ascertaining effects of phosphatase inhibitors on PI3K-Akt pathway, phosphorylation of Akt was analyzed by western blotting according to the method in the previous report [18]. For analysis of western blotting, it was hard to handle non-growing oocytes in the treatment process, because of their too small size. Instead of isolated non-growing oocytes, from newborn pups the ovaries that consist mostly of non-growing oocytes were subjected to organ culture in each experiment and applied to western blotting. Four to eight cultured ovaries in each were applied in 2 x sodium dodecyl sulfate (SDS) sample buffer, 0.5 M Tris-HCl (pH 6.8), 10% (v/v) 2-mercaptoethanol and 20% (v/v) glycerol. The lysates were separated by electrophoresis and transferred onto Immobion membranes (Millipore Japan, Tokyo, Japan). Membranes were incubated overnight at 4°C with an antibody (Cell Signaling Technology, Inc., MA, USA) (1:1,000) against Ser 473- phosphorylated Akt that is revealed as a marker of phosphorylation of Akt in PTEN- knockout mice [10]. The detection with peroxidate-conjugated Affini Pure Goat Anti-rabbit IgG was visualized using ECL Plus Western Blotting Detection System (Amersham Bioscience Japan, Tokyo, Japan).

### Statistical analysis

Data for average diameters of the oocytes were compared using one-way ANOVA with Fisher protected least significant difference (PLSD) by StatView software (Abacus Concepts, Inc., CA, USA), while data for *in vitro* follicle activation and survival rates were analyzed using chi-square test. Differences were considered significant at a level of *P* < 0.05.

## Results

**Figure 1:**
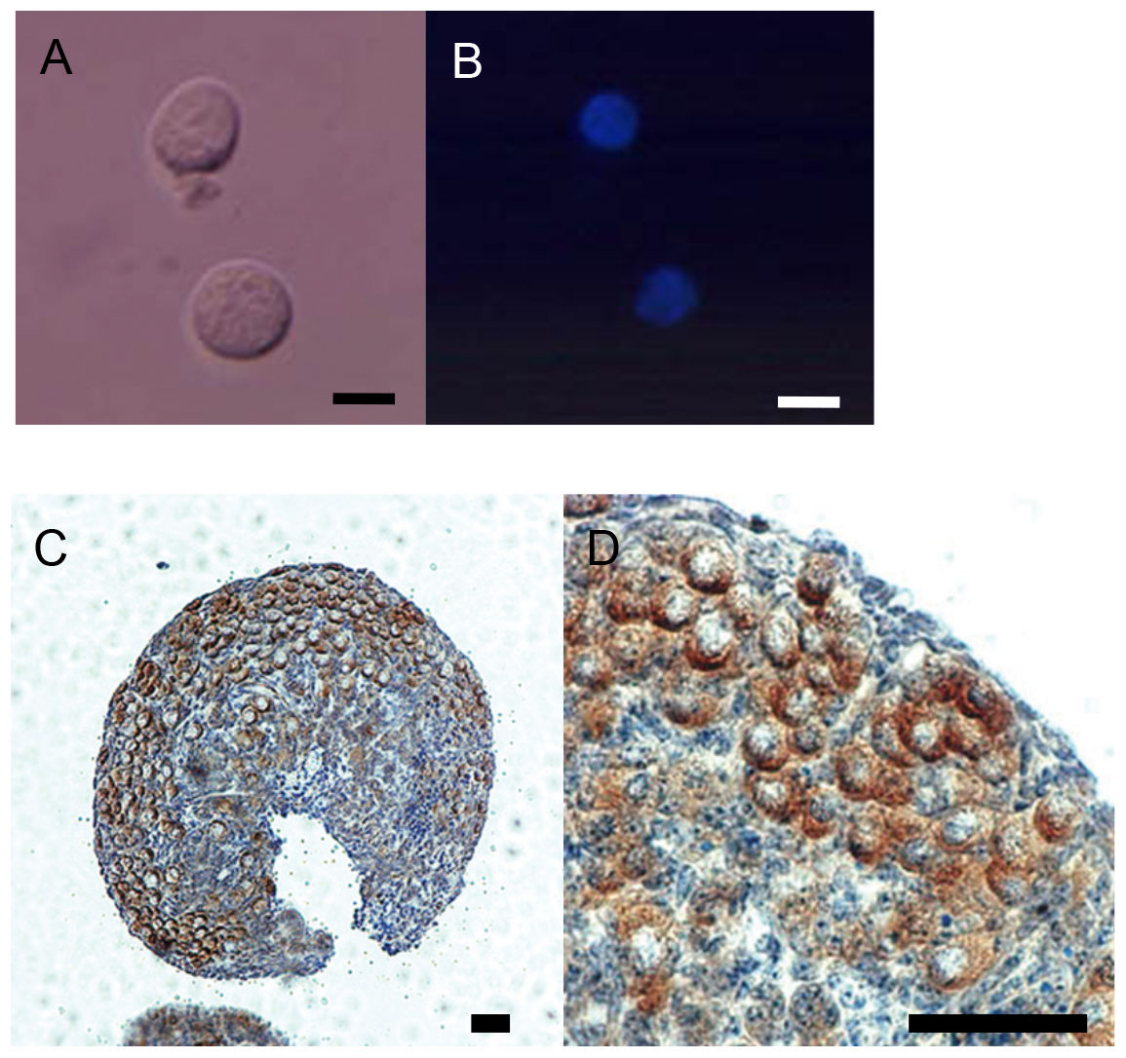
Microphotographs of non-growing oocytes. (A, B) Bright and fluorescent with Hoechst 33342 stain fields. The oocytes were isolated from the ovaries of newborn pups at day 0 of delivery. Bar=10 µm. (C, D; a higher magnification of C) The ovary subjected to immunostatining for MVH. The ovary is occupied by primary oocytes that are MVH positive in the oocyte nests. Bar=50 µm.

Histological examination to ascertain the status of non-growing primary oocytes in the neonatal ovary revealed that non-growing oocytes occupied mostly within the oocyte nests in the ovaries at day 0 of delivery (Figure 1.C and D), and that the ovaries at day 4 of delivery had mainly primordial follicles with non-growing oocytes (data not shown). Hoechst staining of isolated non-growing oocytes from the ovaries at day 0 of delivery showed that each of them had a nucleus in center of its cytoplasm but not surrounded by granulosa cells (Figure 1 A and B). 

**Figure 2:**
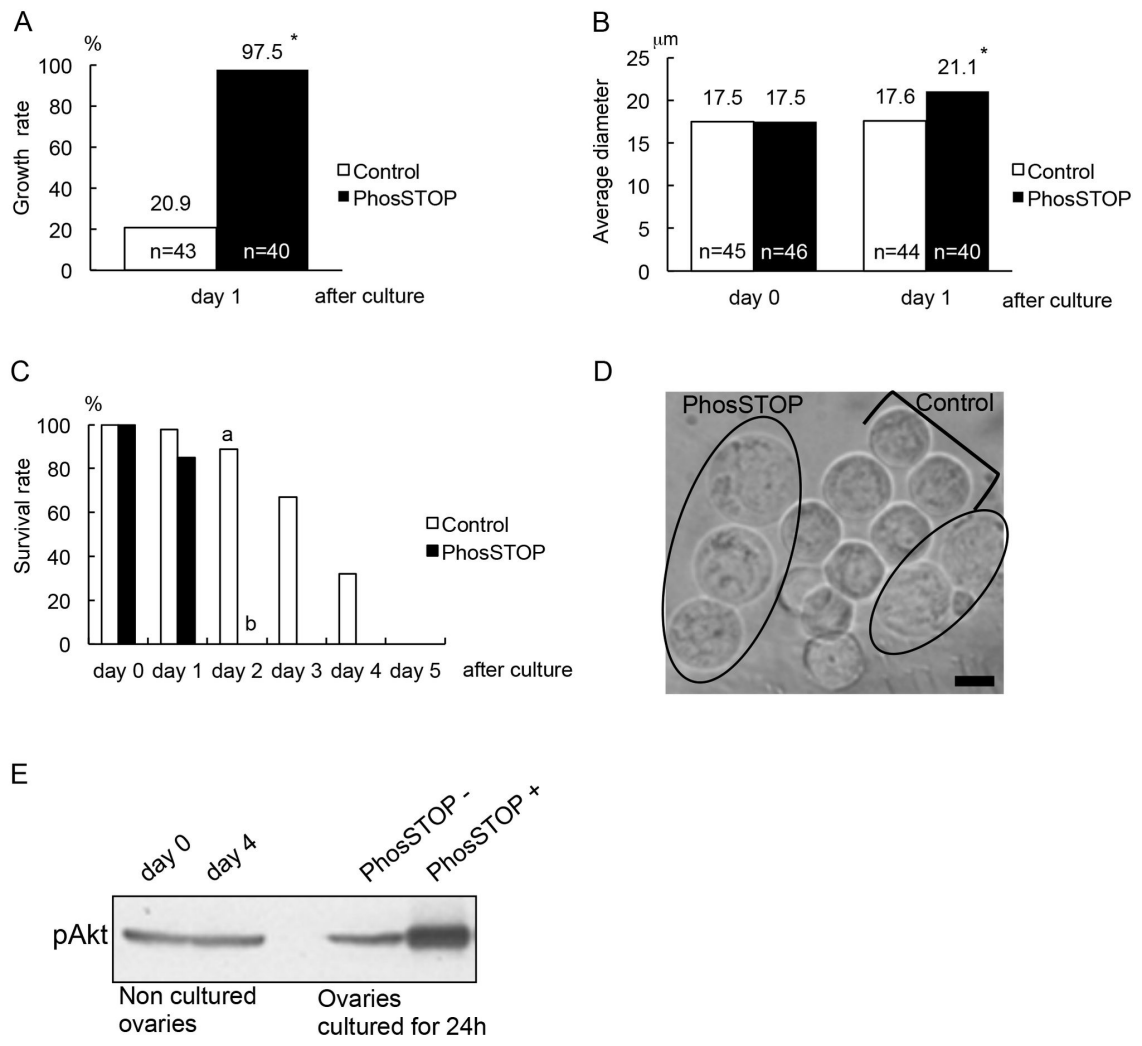
*In* *vitro* growth initiation of non-growing oocytes in PhosSTOP incorporated culture. (A) *In* *vitro* growth rates and (B) average diameters of non-growing oocytes 24 hours after culture both were significantly higher in PhosSTOP group compared to those in the control (*P<0.05). (C) The non-growing oocytes in the control group showed survival up to day 4 of culture, while in the PhosSTOP group they did not. There was a significant difference between two groups (a^,b^ *P*<0.05). (D) Microphotograph of non-growing oocytes in the PhosSTOP and control groups after 24 hours of culture. The oocytes in both groups were together transferred in single culture dish for comparing their figures in the photograph. The circles show the oocytes in the PhosSTOP group and the square brace shows ones in the control. Bar=10 µm. (E) Western blotting of phosphorylated Akt shows strongly detection in the specimen from the ovaries cultured with PhosSTOP.

In experiment 1, the isolated non-growing oocytes were divided to two culture groups: no additive (control) and phosphatase inhibitor (PhosSTOP) incorporated culture media. As a result, after 24 h of culture, *in vitro* follicle activation rate in the PhosSTOP group was significantly (*P*<0.05) higher than that in the control group (97.5 and 20.9%, respectively) (Figure 2 A). Morphology of the oocytes after culture in the PhosSTOP group showed obviously enlargement compared to those in the control (Figure 2 B and D); their diameters shown as mean ± SE were 21.1±0.40 and 17.5±0.18 μm respectively, indicating significant difference between them (*P*<0.05), while PhosSTOP resulted in a reverse effect on survival rate (Figure 2 C). In addition, western blotting of phosphorylated Akt showed enhanced expression in the PhosSTOP group compared to the control and also to fresh ovaries at day 0 and day 4 of delivery (Figure 2 E). 

**Figure 3:**
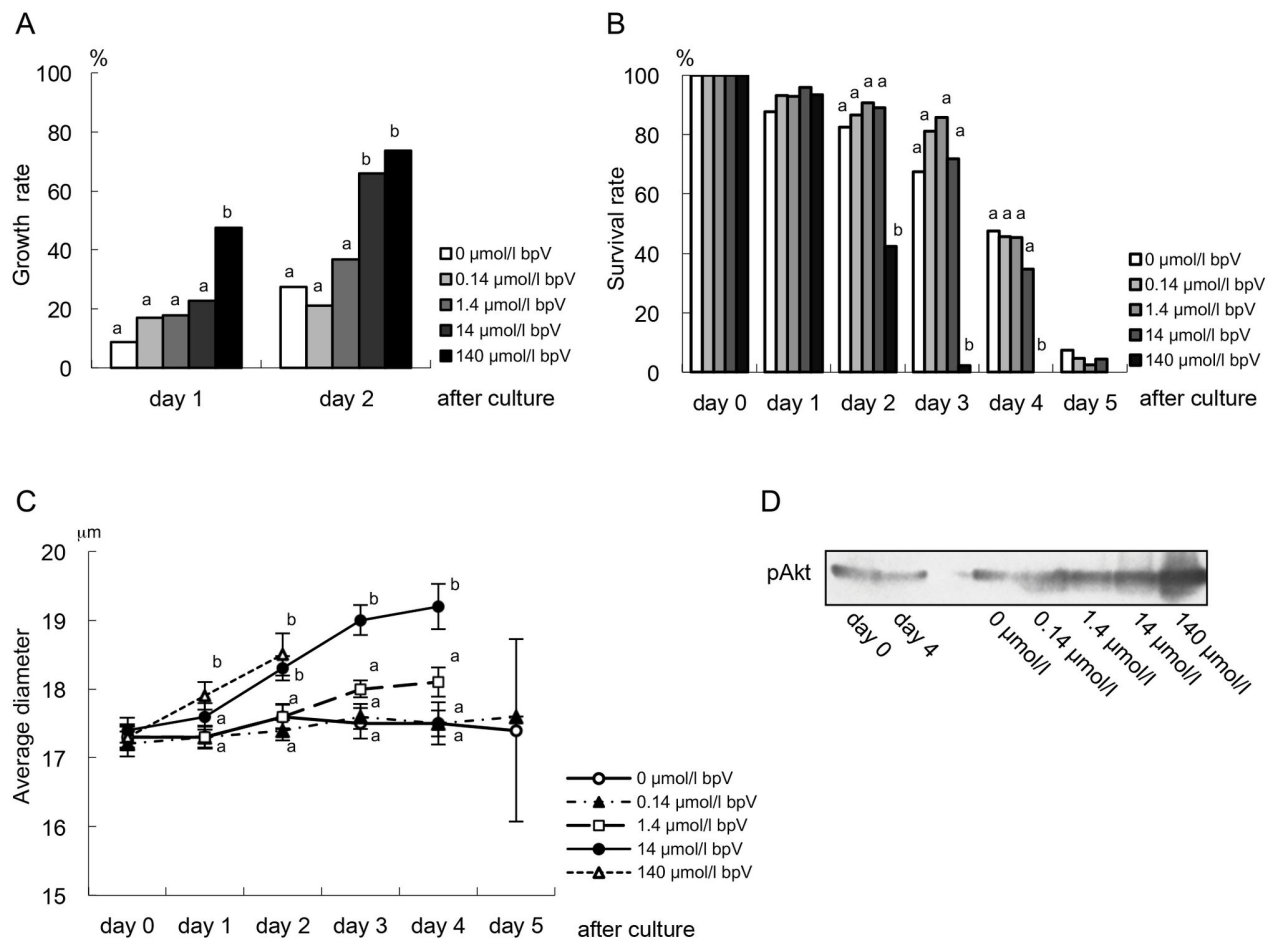
*In* *vitro* growth initiation of non-growing oocytes in PTEN specific inhibitor, bpV incorporated culture. (A) *In* *vitro* growth rates trended increase dependent on concentration of bpV, and were significantly higher in 14 and 140 µmol/l bpV groups (a^,b^ *P*<0.05) (See Table S1 in detail). (B) The oocytes in 0, 0.14, 1.4 and 14 µmol/l bpV groups showed survival up to day 4 of culture, while in the 140 µmol/l bpV group most of them survived during only two days after culture. (C) Average diameters of the oocytes in 14 and 14 µmol/l bpV cultured groups increased significantly during culture. (D) Western blotting of phosphorylated Akt shows strongly detection in the specimens from the ovaries cultured with bpV for 24 hours dependent on bpV concentration.

In experiment 1, although *in vitro* growth of non-growing oocytes increased in the PhosSTOP group, survival rate decreased, perhaps because of detrimental effect of PhosSTOP that affects not only PTEN but also other phosphatases. In experiment 2, therefore, we demonstrated effect of a specific inhibitor of PTEN on growth initiation of non-growing oocytes. The oocytes were cultured in 0 to 140 μmol/l bpV incorporated media. After culture, *in vitro* growth rates increased in the bpV incorporated groups dependent on bpV concentration; the rates at day 2 of culture were significantly higher (*P*<0.05) in the 14 and 140 μmol/l bpV incorporated groups (65.9 and 73.7%, respectively) compared to those in the 0, 0.14 and 1.4 μmol/l bpV incorporated groups (24.2, 21.1 and 36.8 %, respectively) (Figure 3 A). Average diameters of the oocytes increased gradually or constantly in the 14 and 140 μmol/l bpV incorporated groups: 18.3±0.18 and 18.5±0.30 μm, respectively, at day 2 of culture, showing significantly higher (*P*<0.05) compared to those in the 0, 0.14 and 1.4 μmol/l bpV incorporated groups (17.6±0.17, 17.4±0.15 and 17.6±0.18 μm, respectively) (Figure 3 C). In addition, western blotting of phosphorylated Akt showed enhanced expression by a dependent manner of concentration in the bpV groups compared to the control (Figure 3 D). Different from the results in experiment 1, the oocytes in the bpV incorporated groups showed survival at day 5 of culture; survival rates at day 2 of culture in the 0.14, 1.4 and 14 μmol/l bpV incorporated groups (86.4, 90.5 and 89.1%, respectively) were same as that in the control (82.5%) (Figure 3 B).

**Figure 4:**
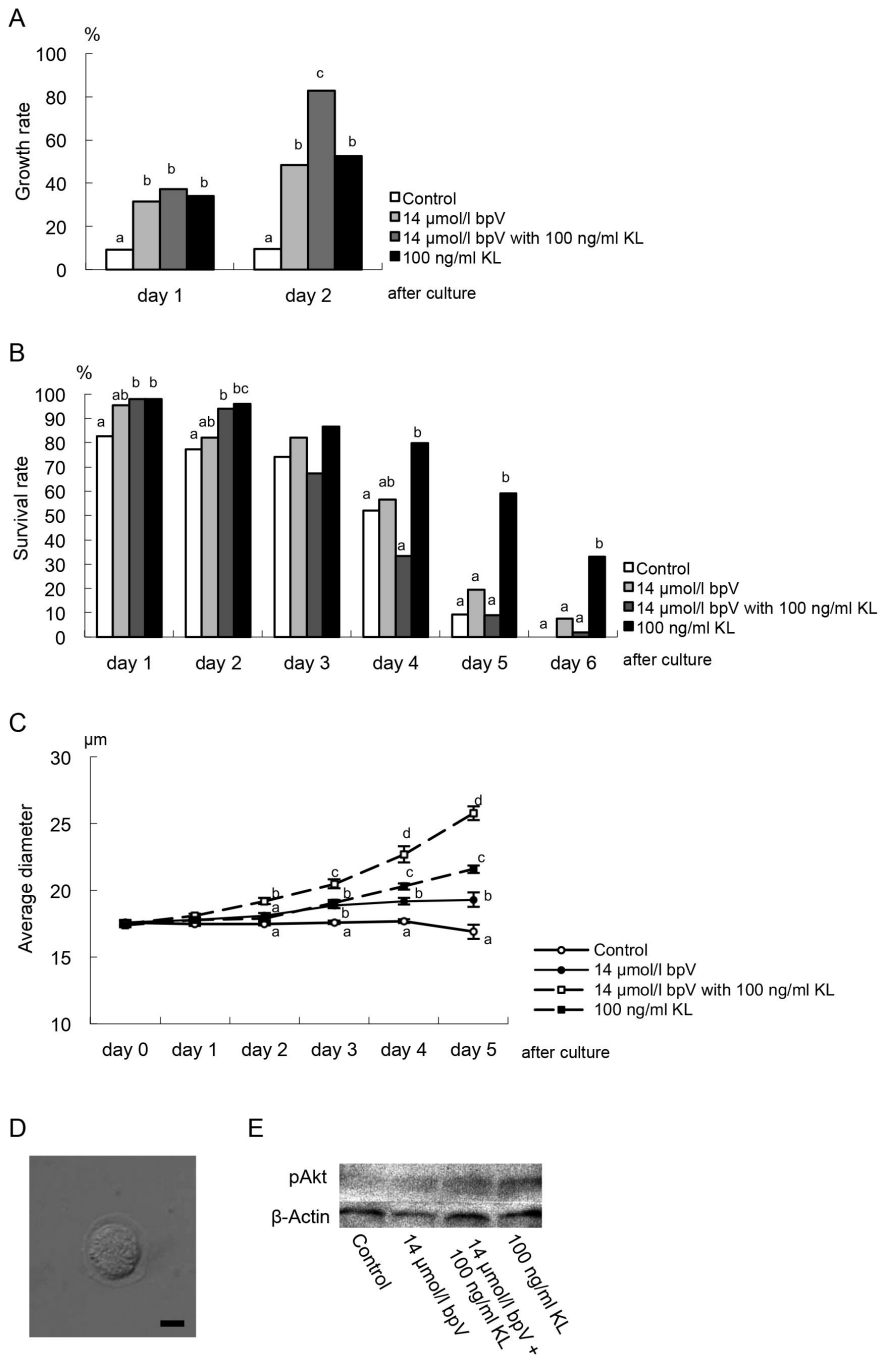
*In* *vitro* growth of non-growing oocytes in bpV and Kit Ligand incorporated culture. (A) *In* *vitro* growth rates in 14 µmol/l bpV with 100 ng/ml KL culture group increased significantly compare to other groups (a^,b^ *P*<0.05) (See Table S2 in detail). (B) Among culture groups, survival rates in both 14 µmol/l bpV with 100 ng/ml KL and only 100 ng/ml KL groups showed significantly higher compared to others throughout first 3 days of culture, and afterward the latter group sustained higher survival rate. (C) Average diameters of the oocytes in 14 µmol/l bpV with 100 ng/ml KL group greatly increased compared to another groups 5 days after culture. (D) Microphotograph of typical morphology in growing oocytes that formed zona pellucidae 6 days after culture in 14 µmol/l bpV with 100 ng/ml KL group. Bar=10 μm. (E) Western blotting of phosphorylated Akt shows detection in the ovaries cultured in bpV and KL incorporated medium for 24 hours.

Based on the results in experiment 1, 2 and the preliminary test for effect of KL, in experiment 3, we demonstrated synergic effect of bpV and KL in culture medium on growth initiation and subsequent growth of non-growing oocytes. In this case, the oocytes were cultured with both the bpV and KL incorporated medium. As a result, *in vitro* growth rate at day 2 of culture increased significantly (*P*<0.05) in the 14 μmol/l bpV plus 100 ng/ml KL group (Figure 4 A). Viability of oocytes did not necessarily show difference among the groups during first 3 days of culture, and later was significantly (*P*<0.05) higher in the KL incorporated group than those in the other groups (Figure 4 B). In addition, western blotting of phosphorylated Akt showed enhanced expression in the KL groups compared to control. After culture of 5 days, the oocytes in the 14 μmol/l bpV only, 14 μmol/l bpV plus 100 ng/ml KL, and 100 ng/ml KL groups showed significantly (*P*<0.05) larger growth (19.3±0.55, 25.8±0.53 and 21.6±0.29 μm in diameter, respectively) with formation of zona pellucida compared with that in the control (no additive) (16.9±0.53 μm) (Figure 4 C, D). Considered that non-growing primary oocytes are present as many as several ten thousands in an ovary, even only a few percentage of growing rate can supply plentiful number of oocytes available for further production of eggs.

## Discussion

During a process for forming germ cells in mice, primordial germ cells differentiate in the fetal ovary into oogonia that prior to folliculogenesis differentiate non-growing primary oocytes at germinal vesicle stage as germline cysts in the ovary [3,19,20]. After birth, from the cysts primordial follicles start to be formed as single non-growing oocyte is surrounded by a few of flatten granulosa cells, and are recruited into growing follicle pool [21–23]. Follicle growth initiation or follicle activation from primordial follicle pool can be substantially evaluated by growth initiation of primary oocytes included, which is termed as the entry of primordial follicles into primary follicle stage [1,3]. In the present study, to demonstrate *in vitro* induction of oocyte growth by phosphates inhibitor, we isolated and applied non-growing oocytes that are occupied in neonatal ovaries, showing first as we know that phosphatase inhibitor, especially to PTEN can stimulate *in vitro* growth initiation of isolated non-growing primary oocytes and that more effectively concomitant with KL the oocytes can be grown with formation of their surrounding zona pellucidae by mediation of PI3K-Akt signaling. 

In general, PI3K-Akt signaling is first caused by binding survival signals such as insulin-like growth factor (IGF) and KL to a receptor tyrosine kinase (RTK) on the extracellular domain. Secondly, activated RTK activate PI3K to produce phosphatidylinositol 3,4,5-trisphosphate (PI(3–5)P_3_) that phosphorylates phosphoinositide-dependent protein kinase 1 (PDK1) and Akt, respectively, and then activated Akt phosphorylates various target proteins to prevent apoptosis and enhance cell survival and growth [13,24,25]. Recent study in *Pten* (phosphatase and tensin homolog deleted on chromosome 10) -knockout mice [10] has revealed that this phosphatase acts as an inhibitory factor in follicle activation of primordial follicle pool. Lipid phosphatase PTEN dephosphorylates the 3 position of the inositol ring of inositol phospholipids as a major negative regulator of PI3K [26–28]. In experiment 1 of this study, we revealed that incorporation of phosphatase inhibitor, PhosSTOP in culture medium promoted *in vitro* growth of isolated non-growing oocytes. Since western blotting showed enhanced expression of phosphorylated Akt, *in vitro* follicle activation may be caused by inhibiting PTEN that is committed in the intra-oocyte PI3K-Akt signaling cascade. The survival rate of non-growing oocyte, however, was decreased sharply after 24 hours of culture with PhosSTOP, because this substance is not a specific inhibitor of PTEN, and so can affect not only PTEN but also other phosphatases that need oocyte survival. Therefore, in experiment 2 of this study, we demonstrated effect of bpV on *in vitro* growth and survival of non-growing oocytes. The bpV known as an inhibitor of PTPase has been reported to specifically suppress PTEN [14,29,30]. With incorporation of bpV into culture medium, we found increases in both *in vitro* growth rate and average diameter of oocytes with extension of survival up to 5 days of culture. In the previous reports, the extent of primordial follicle activation differs significantly in different species and with different types of ovarian tissues used [1,7,31–35]. Usually, whole ovaries from neonatal rats or mice are used for *in vitro* culture, whereas in species with large ovaries, like humans and cattle, only cortical tissues that are rich in primordial follicles are used. Most of the primordial follicles in bovine and baboon cortical slices become activated during *in vitro* culture [36], whereas only limited numbers of primordial follicles are activated in cultured mouse ovaries [3,8,9]. Different from culture of ovarian tissues in the previous study, isolation of non-growing oocytes or primordial follicles seems to supply better materials for *in vitro* culture, because of less difficulty from tissues and more utility even in large ovaries. Although the present study demonstrated isolated non-growing primary oocytes, the culture method developed could be useful for applying to *in vitro* culture of primordial follicles, because they contain non-growing oocytes. Since non-growing oocytes are stored in vivo as individual follicles and grown accompanied by follicle development, it is known that oocyte growth is sustained by its surrounding cells (granulosa cells). The relationship between oocyte and accompanied cells, however, differs according to follicle developmental stage, in which initial growth of non-growing oocytes is sustained by humoral factors rather than granulosa cells. Furthermore, the present study shows not merely possibility of culture for non-growing primary oocytes, but future culture without supporting cells, which can allow us use of female germ cells in both small and large animals. Further, a method of culture of non-growing primary oocytes could supply us plentiful resources of female germ cells.

As a regulator of PI3K, while the phosphatase PTEN negatively regulates PI3K-Akt signaling, many studies have shown that this signaling is activated by KL-Kit in the hematopoietic, melanogenic, and gametogenic cell lineages, and also in mast cells [2]. KL and its receptor Kit are encoded by the *W* and *Sl* loci, and KL-Kit mutation has shown defect in primordial germ cell development and infertility in female mice [3,37,38]. In addition, the roles of KL-Kit have been known to promote spontaneous growth of primordial follicles to the primary follicle stage and to have effect during early folliculogenesis [39,40]. Therefore, KL is an important growth factor for primordial follicles to promote to primary follicle transition [41–44]. In experiment 3 of this study, we applied bpV, a specific inhibitor to PTEN and KL together to culture medium. *In vitro* growth rate and average diameter of non-growing oocytes in bpV and KL incorporated group showed significantly increases compared to other groups, showing their synergic effect on *in vitro* growth of these oocytes. Considered together that the effects on *in vitro* growth rate and average diameter in bpV group were same as those in KL group during the first 2 days of culture, it may be suggested that once the preceding effect on *in vitro* growth initiation of oocytes by inhibition of PTEN does work, the following growth could be stimulated by KL in culture. In addition, it has been known that zona pellucidae that are not formed yet in primordial follicles are formed from mucopolysaccharide and proteins secreted by oocytes during transit from primary to secondary follicles [2,3]. Hence, in the present study the resultant formation of zona pellucida in isolated non-growing oocytes cultured with bpV and KL may be evaluated as the oocytes have grown with normal function. Moreover, this finding may be the first about zona pellucida formation in *in vitro* culture of isolated non-growing oocytes.

In conclusion, we show first as we know that phosphatase inhibitor, especially PTEN can stimulate *in vitro* growth of isolated non-growing primary oocytes and that more effectively concomitant with KL the oocytes can be grown with formation of their surrounding zona pellucidae. We believe that the culture method developed in this study may be contributed in developing a technique for *in vitro* folliculogenesis in small and large animals, which also can lead to establish a system for cell-free culture of non-growing primary oocytes.

## Supporting Information

Table S1 ***In vitro* growth rate of non-growing oocytes after culture with bpV.**
(DOCX)

Table S2 ***In vitro* growth rate of non-growing oocytes after culture with either of bpV, bpV and KL or KL.**
(DOCX)
