# Supplementary material for: Incorporation of Phosphatase Inhibitor in Culture Prompts Growth Initiation of Isolated Non-Growing Oocytes
Source: PLoS One. 2013 Nov 4;8(11):e77533. doi: 10.1371/journal.pone.0077533 (PMC3817191; doi:10.1371/journal.pone.0077533)
Supplement: Table S1 — In vitro growth rate of non-growing oocytes after culture with bpV. (DOCX) [file pone.0077533.s001.docx]

**Table S1. *In vitro* growth rate of non-growing oocytes after culture with bpV**

|  |  | **day 1** | | **day 2** | |
| --- | --- | --- | --- | --- | --- |
| **Concentration of bpV (μmol/l)** | **No. of oocytes used** | **No. of oocytes survived** | **No. of oocytes grown** | **No. of oocytes survived** | **No. of oocytes grown** |
| 0 | 40 | 35 | 3 (8.6) *^a^ | 33 | 9 (27.3) ^a^ |
| 0.14 | 44 | 41 | 7 (17.1) ^a^ | 38 | 8 (21.1) ^a^ |
| 1.4 | 42 | 39 | 7 (17.9) ^a^ | 38 | 14 (36.8) ^a^ |
| 14 | 46 | 44 | 10 (22.7) ^a^ | 41 | 27 (65.9) ^b^ |
| 140 | 45 | 42 | 20 (47.6) ^b^ | 19 | 14 (73.7) ^b^ |

The data were collected from at least three experiments.

* The percentage out of No. of oocytes survived.

^a, b^ There were significantly differences between different characters (*P*<0.05).
