# Supplementary material for: Incorporation of Phosphatase Inhibitor in Culture Prompts Growth Initiation of Isolated Non-Growing Oocytes
Source: PLoS One. 2013 Nov 4;8(11):e77533. doi: 10.1371/journal.pone.0077533 (PMC3817191; doi:10.1371/journal.pone.0077533)
Supplement: Table S2 — In vitro growth rate of non-growing oocytes after culture with either of bpV, bpV and KL or KL. (DOCX) [file pone.0077533.s002.docx]

**Table S2.** ***In vitro* growth rate of non-growing oocytes after culture with either of bpV, bpV and KL or KL**

|  |  | day 1 | | day 2 | |
| --- | --- | --- | --- | --- | --- |
| Treatments | No. of oocytes used | No. of oocytes survived | No. of oocytes grown | No. of oocytes survived | No. of oocytes grown |
| Control | 40 | 33 | 3 (9.1)* ^a^ | 31 | 3 (9.7) ^a^ |
| 14 μmol/l bpV | 40 | 38 | 12 (31.6) ^b^ | 33 | 16 (48.5) ^b^ |
| 14 μmol/l bpV with 100 ng/ml KL | 44 | 43 | 16 (37.2) ^b^ | 41 | 34 (82.9) ^c^ |
| 100 ng/ml KL | 42 | 41 | 14 (34.1) ^b^ | 40 | 21 (52.5) ^b^ |

The data were collected from at least three experiments.

* The percentage out of No. of oocytes survived.

^a, b^ There were significantly differences between different characters (*P*<0.05).
